# Delta-Aminolevulinic Acid-Mediated Photodiagnoses in Surgical Oncology: A Historical Review of Clinical Trials

**DOI:** 10.3389/fsurg.2019.00045

**Published:** 2019-09-04

**Authors:** Joseph F. Georges, Amber Valeri, Huan Wang, Aaron Brooking, Michael Kakareka, Steve S. Cho, Zein Al-Atrache, Michael Bamimore, Hany Osman, Joseph Ifrach, Si Yu, Carrie Li, Denah Appelt, John Y. K. Lee, Peter Nakaji, Kristin Brill, Steven Yocom

**Affiliations:** ^1^Department of Neurosurgery, Philadelphia College of Osteopathic Medicine, Philadelphia, PA, United States; ^2^Department of Neurosurgery, Cooper University Healthcare, Philadelphia, PA, United States; ^3^School of Medicine, Cooper Medical School of Rowan University, Camden, NJ, United States; ^4^Perelman School of Medicine, University of Pennsylvania, Philadelphia, PA, United States; ^5^Department of Neurosurgery, Hospital of the University of Pennsylvania, Philadelphia, PA, United States; ^6^School of Medicine, Philadelphia College of Osteopathic Medicine, Philadelphia, PA, United States; ^7^Wellman Center for Photomedicine, Massachusetts General Hospital, Boston, MA, United States; ^8^Department of Neurosurgery, St. Joseph's Hospital and Medical Center, Barrow Neurological Institute, Phoenix, AZ, United States; ^9^Department of Surgery, MD Anderson Cancer Center at Cooper Health Systems, Camden, NJ, United States

**Keywords:** fluorescence, 5ALA, protoporphyrin IX, surgery, neurosurgery, glioma

## Abstract

Fluorescence imaging is an emerging clinical technique for real-time intraoperative visualization of tumors and their boundaries. Though multiple fluorescent contrast agents are available in the basic sciences, few fluorescence agents are available for clinical use. Of the clinical fluorophores, delta aminolevulinic acid (5ALA) is unique for generating visible wavelength tumor-specific fluorescence. In 2017, 5ALA was FDA-approved for glioma surgery in the United States. Additionally, clinical studies suggest this agent may have utility in surgical subspecialties outside of neurosurgery. Data from dermatology, OB/GYN, urology, cardiothoracic surgery, and gastrointestinal surgery show 5ALA is helpful for intraoperative visualization of malignant tissues in multiple organ systems. This review summarizes data from English-language 5ALA clinical trials across surgical subspecialties. Imaging systems, routes of administration, dosing, efficacy, and related side effects are reviewed. We found that modified surgical microscopes and endoscopes are the preferred imaging devices. Systemic dosing across surgical specialties range between 5 and 30 mg/kg bodyweight. Multiple studies discussed potential for skin irritation with sun exposure, however this side effect is infrequently reported. Overall, 5ALA has shown high sensitivity for labeling malignant tissues and providing a means to visualize malignant tissue not apparent with standard operative light sources.

## Introduction

Surgeons have utilized light to better visualize their surgical fields since antiquity. Fluorescence, a relatively new discovery, has been studied since the early-to-mid twentieth century as a means for providing better contrast of structures during surgery. Reports from the late twentieth century showing fluorescence contrast could improve intraoperative visualization of tumors during surgery has fueled a renewed interest in further developing clinical fluorescence imaging techniques.

In the United States, few agents are clinically approved for generating intraoperative fluorescence contrast (1). Though novel agents are in various stages of clinical trials, the three agents currently approved for clinical use are fluorescein, indocyanine green, and delta-aminolevulinic acid (5ALA), and of these, only 5ALA has a specific FDA clinical indication for CNS use (2). Of these, 5ALA is also the only agent that produces intracellular tumor-specific fluorescence. Delta-aminolevulinic acid generates this fluorescence by causing tumor-specific accumulation of the fluorescent molecule protoporphyrin IX (PpIX).

Delta-aminolevulinic acid is produced by the condensation of succinate and glycine, and was first reported in a Nature article in 1953 (3, 4). Studies with radiolabeled carbon showed this molecule was involved in porphyrin synthesis (3). Studies during the 1950s and 1960s focused on the role of 5ALA in the heme synthesis pathway (5, 6). The first human studies with 5ALA were conducted in 1956, and were devised to study 5ALA metabolism. In these studies, 5ALA was given orally (7). Skin sensitivity, a well-known complication of 5ALA, was first reported in 1956 (7). This phenomenon occurred 2–11 h after 5ALA administration.

Porphyrin-mediated fluorescence was first reported in the late nineteenth century. However, the potential role porphyrins would have in tumor visualization would not be reported until half a century later in lab-based studies. The earliest clinical studies of 5ALA-mediated tumor visualization were reported for dermatology and urology in 1990 and 1994, respectively (8–10). Throughout the remaining twentieth century and early twenty first century, clinicians and scientists continued to evaluate the role of 5ALA-mediated fluorescence for intraoperative photodiagnosis of neoplasms in neurosurgery, head and neck surgery, cardiothoracic surgery, gastrointestinal surgery and OB/GYN.

This review provides a history of notable findings for 5ALA-mediated photodiagnosis across surgical subspecialties. The information is derived from a PubMed search of all English language clinical trials published between 1950 and 2018. Basic science and animal studies are excluded from this review.

## Neurosurgery

### First Application of 5ALA in Neurosurgery

The first study investigating 5ALA in neurosurgery was published by Stummer et al. (11). In this seminal study, Stummer administered 10 mg/kg of 5ALA orally to 9 patients with high-grade gliomas (HGG) 3 h prior to induction of anesthesia. Using a 375–440 nm bandpass filter, Stummer was able to provide blue-light excitation. A long-pass filter >455 nm blocked the reflected excitation light and allowed the emitted protoporphyrin IX fluorescence to be visualized. From the 9 patients, Stummer took a total of 89 biopsies; in these specimens, 5ALA fluorescence demonstrated 85% sensitivity, 100% specificity, and 90% accuracy. The major contributors to the false-negatives were areas of low-density infiltrates of malignant cells and necrotic areas of the tumors. At the 10 mg/kg dose, no adverse effects were recorded. This study, while small, established the utility of 5ALA as an intraoperative imaging agent and began a new era for fluorescence-guided neurosurgery studies.

### Landmark 5ALA Studies in Neurosurgery

Since the first paper in 1998, Stummer et al. and other groups have continued to investigate various aspects of 5ALA in neurosurgery. In 2000, Stummer et al. published a study in a larger cohort of 52 patients with HGGs, this time stratifying fluorescence subjectively into strong, vague, and none (12). The study demonstrated again that 5ALA was highly sensitive and specific for neoplasm and that residual fluorescence after standard resection predicted subtotal resection, seen on postoperative MRI as residual enhancement. The most impactful clinical neurosurgery 5ALA study was the 2006 randomized, controlled, multi-center trial by Stummer et al. (13). In the study, 270 HGG patients were randomized to either fluorescence-guided surgery with 5ALA or conventional surgery with white-light alone. Importantly, the study demonstrated that those in the 5ALA arm achieved gross total resection (GTR) at a much higher rate (65% vs. 36%, *p* < 0.0001) and had significantly higher progression-free survival (PFS) at 6 months (41% vs. 21.1%, *p* = 0.0003) without significant changes in postoperative neurologic deficits. Although the study was underpowered to demonstrate effects on overall survival, the results were nonetheless encouraging. In 2014, Coburger further demonstrated that 5ALA was more accurate than intraoperative MRI for detecting neoplasm at the infiltration zone, establishing 5ALA as both a more cost-effective and superior alternative (14).

### Appropriate 5ALA Dose and Route of Administration

The current accepted dose for 5ALA administration in neurosurgery is an oral dose of 20 mg/kg bodyweight ~3 h before induction of anesthesia, translating to roughly 4–5 h before tumor exposure. In Stummer et al.'s first study in 1998, the dose used was 10 mg/kg. Two later studies investigated different doses of 5ALA in neurosurgery: a 2017 study by Stummer et al. (15) and another 2017 study by Eljamel et al. (16). Stummer et al. studied the efficacy of 0.2, 2, and 20 mg/kg in a total of 21 patients with HGGs and demonstrated that a 10-fold increase in dosage from 2 to 20 mg/kg yielded only a 4-fold increase in signal. They concluded that a dose higher than 20 mg/kg was unlikely to yield significant benefits. Eljamel et al. on the other hand, investigated doses from 10 to 50 mg/kg in 10 mg/kg increments in 19 patients with HGGs. They concluded that a dose of up to 50 mg/kg was safe in patients and suggested that higher doses of 5ALA may increase tumor fluorescence, although their results were not statistically significant in their small sample population. Considering these results, most groups, including Stummer et al. have transitioned to 20 mg/kg, which seems to be both safe and effective.

In terms of route of administration, 5ALA has always been administered orally for neurosurgical patients. Preclinical studies, and studies in healthy patients, have established that oral 5ALA is rapid and effective (17). Thus, changes to the route of administration have not been considered for 5ALA in neurosurgery.

### Imaging 5ALA Fluorescence in Neurosurgery

**Figure 1:**
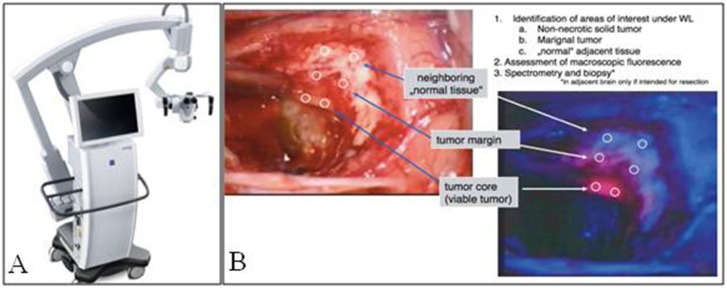
Fluorescence imaging in neurosurgery. **(A)** Zeiss Pentero fluorescence surgical microscope used for intraoperative imaging. **(B)** Brightfield illumination compared to PpIX fluorescence from a malignant glioma, note fluorescence signal of the tumor compared to tumor margin and normal adjacent brain [Intraoperative images courtesy of Stummer et al. (15)].

Neurosurgeons have relied heavily on surgical microscopes for intracranial surgeries since the 1950s (18). Hence, the majority of 5ALA visualization in neurosurgery has been performed with surgical microscopes (Figure 1A). In general, add-on modules are attached to existing microscopes to achieve blue-light excitation. Initially, when Stummer et al. first described their experience with 5ALA, a 375–440 nm bandpass filter was placed in front of the Xenon light, which provided UV and blue-light excitation. A long-pass >455 nm filter was used to block the reflected excitation light, in order to better detect the red PpIX fluorescence. This setup was used by Stummer's group and other groups until the mid-2000's, when Zeiss introduced its Blue400 module, which uses a 400–410 nm filter for excitation and 620–710 nm filter for emission and can rapidly toggle between white-light and blue-light illumination. Band pass emission filters at 620–710 nm were initially utilized to specifically visualize the peak red emission of PpIX; however, most surgeons found it difficult to operate with red light only. Hence, emission filters in the next generation of microscopes were changed from the narrow band pass filter to a 444 nm long pass filter. Although the field is illuminated only with blue light, autofluorescence in green and mild yellow provide the surgeon with illumination of surrounding background structures while resecting pink/red fluorescent tissue (Figure 1B). Leica has recently offered its FL400 module (380–430 nm excitation filter with a 444 nm long pass emission filter), which, similar to the Zeiss module, provides intraoperative visualization of PpIX.

Although exoscopes are a relatively new addition to the neurosurgeons' armamentarium, at least one group has attempted 5ALA visualization using an exoscope. Piquer et al. demonstrated that a commercial exoscope fitted with a 380–430nm excitation filter and >444 nm emission filter could be used to visualize 5ALA fluorescence reliably in the operating room (19, 20). Their study remains one of few studies to visualize 5ALA fluorescence without standard microscope equipment.

### Current Status of 5ALA

In June 2017, 5ALA was FDA-approved as an intraoperative visualizing agent for patients with HGGs. Although 5ALA has been difficult for U.S. neurosurgeons to access outside of research studies, that is slowly changing as of April 2019 (2).

### Future Applications of 5ALA Fluorescence in Neurosurgery

Though most clinical 5ALA studies have focused on patients with HGGs, some groups have investigated its application in other intracranial tumors. For instance, Widhalm et al. (21, 22) demonstrated that non-enhancing gliomas are poor targets for 5ALA and Valdes et al. (23) concluded that conventional 5ALA imaging was of limited use in patients with low-grade gliomas. On the other hand, Valdes et al. (24) and Coluccia et al. (25) investigated the utility of 5ALA in meningiomas and concluded that most meningiomas (80 and 94%, respectively) demonstrated PpIX fluorescence. Similarly, Eljamel et al. concluded that 5ALA fluorescence was useful in pituitary adenomas of various subtypes (26). These studies offer encouraging evidence that 5ALA fluorescence may be applicable to other intracranial tumors and may further help neurosurgeons visualize tumors in the operating room.

Technological advances are improving clinical 5ALA fluorescence detection. Thus far, most neurosurgeons have relied on qualitative grading of PpIX fluorescence (i.e., strong, vague, none), which is limited in objectivity and sensitivity. Multiple groups have demonstrated that quantitative grading of fluorescence, achieved with an intraoperative spectrometry probe, increases the sensitivity and accuracy of 5ALA fluorescence (23, 24, 27). Furthermore, an important limitation in 5ALA fluorescence is the micron-scale tissue penetration by ultraviolent excitation light, which can hinder sensitive tumor detection. Therefore, to increase depth penetration of excitation light, Roberts et al. recently demonstrated that using red-light excitation (620–640 nm) and a sensitive spectrally-resolved camera to take advantage of 5ALA's second, smaller excitation/emission peak, neoplastic areas could be visualized up to 5 mm below the tissue surface (28).

### Conclusion

Overall, 5ALA has demonstrated utility in increasing GTR rates and PFS in patients with HGGs and may be applicable to other intracranial tumors as well. Along with potential advances in intraoperative visualization techniques, 5ALA may ultimately improve patient outcomes in neurosurgery.

## Urology

### First Application of 5ALA in Urology

The first study to evaluate 5ALA in Urology was published by Kriegmair et al. (9). In this study, an intravesicular application of 1.5 g of 5ALA dissolved in 50 ml of sodium bicarbonate was instilled. Time of exposure of 5ALA ranged from 15 to 360 min. The mean time between instillation and fluorescence cystoscopy was 204 min. Urologic surgery was performed under violet light from a krypton ion laser with 406.7 nm excitation. This provided visualization of red fluorescence from protoporphyrin IX in the urothelium of the bladder to perform biopsies of the bladder wall in 68 patients who were suspected to have bladder cancer. In this study, photodynamic diagnosis utilizing 5ALA fluorescence-directed urothelium biopsy diagnosed bladder cancer with a high sensitivity of 100% and specificity of 68.5%. No serious side effects were observed with intravesicular instillation of 5ALA. This study generated confidence that 5ALA could provide highly sensitive visualization and improved resection of difficult-to-detect lesions, such as carcinoma *in situ* and urothelial dysplasia, resulting in diminished recurrence rates.

### Appropriate 5ALA Dose and Route of Administration

5ALA can be administered via two routes for the diagnosis of urologic malignancies. In early studies, a 1.5 g, 3% solution of 5ALA was given almost exclusively by direct intravesicular instillation 2–3 h before biopsy was performed. In more recent studies, researchers have transitioned to administering a 20 mg/kg body weight oral solution of 5ALA 3–4 h before biopsy is performed. Inoue et al. evaluated the safety and efficacy of 10 mg/kg vs. 20 mg/kg of oral 5ALA in white light vs. fluorescence cystoscopy in a total of 62 patients (29). The rates of carcinoma *in situ* and high grade non-invasive papillary urothelial carcinoma detected only by white light cystoscopy was 4.0 and 0.0%, respectively in the 10 and 20 mg/kg groups vs. 16.0 an 36.1%, respectively in the 10 and 20 mg/kg fluorescence cystoscopy groups. Inoue et al. performed a follow-up study in 2016 which demonstrated that sensitivity increased in a dose-dependent manner with fluorescence cystoscopy, reporting 83.7% at ≧ 10 mg/kg and <15 mg/kg, 86.4% at ≧ 15 mg/kg and <20 mg/kg, and 96.3% at ≧ 20 mg/kg (30).

### Imaging 5ALA Fluorescence in Urology

**Figure 2:**
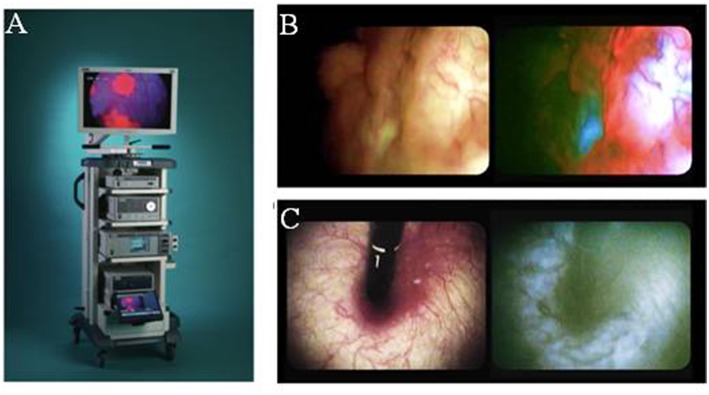
Fluorescence imaging in urology. **(A)** Karl Stortz D-light C used for blue-light cystoscopy as an adjunct to white-light cystoscopy for detection of non-muscle invasive bladder cancer in patients with suspected or known lesion(s). **(B)** White light mode (left side) and blue light mode (right side) images of bladder cancer simultaneously observed using twin mode monitor. Flat lesions show red florescence. **(C)** White light mode (left side) and blue light mode (right side) images of bladder neck using turned back flexible cystoscopy in a vertical direction. No red fluorescence observed [Intraoperative images courtesy of Fukuhara et al. (31)].

In the first study to document the results of 5ALA photodynamic diagnostics in urologic malignancy, Kriegmair et al. utilized a krypton ion laser 406.7 nm excitation. The majority of studies published in this field utilize a xenon arc lamp with a 370–440 nm bandpass filter, with or without long-pass filter to detect the red PpIX fluorescence. A fluorescence cystoscope, sometimes the help of a 0 or 30 telescope is typically employed (Figure 2) (32, 33). Fukuhara et al. published a study in 2015 reporting the utility of a flexible fluorescence-cystoscope with a twin-mode monitor in 5ALA photodynamic diagnosis of bladder cancer (31). In this particular study, a new PDD system consisting of a fluorescence endoscope with a SAFE-3000 processor and flexible cystoscope with a xenon lamp and semiconductor laser. Fluorescence images were observed with a charge coupled device image processor. A twin-mode monitor allowed a white light image and fluorescent image to be visualized simultaneously.

### Adverse Effects of 5ALA

Intravesicular application of 5ALA is overall, well-tolerated with minimal side effects. Multiple studies reported transient urinary urgency, pollakisuria, and alginuresis (34, 35). With the oral administration of 5ALA, patients are often described as having transient hypotension, transaminitis and skin photosensitivity (29, 31, 36).

### Limitations of 5ALA in Diagnosis of Bladder Cancer

Speiss et al. reported in 2006 that fluorescence cystoscopy with 5ALA can have a false-positive rate as high as 40% (37). Possible causes of a false-positive result include: inflammation, urothelial hyperplasia, recent urethral stents, bacteriuria, previous intravesicular therapy within the previous 3 months, and inexperience of the performing Urologist (38, 39).

### Current Status of 5ALA in Urology

The United Stated Food and Drug Administration approved 5ALA for intra-operative photodynamic diagnosis of bladder cancers in 1999. Currently, studies are being conducted regarding the use of a 5ALA ester, hexyl aminolevulinate, to compare efficacy and diagnostic accuracy.

### Conclusion

Overall, 5ALA has demonstrated increased sensitivity in diagnosis of bladder cancers compared to white-light cystoscopy, especially in the case of carcinoma *in situ* and dysplasia. It has shown to improve visualization of surgical margins at resection and to decrease recurrence rates.

## Dermatology

### First Application in Clinical Dermatology

The first study investigating 5ALA in dermatology was published by Kennedy et al. (40). Though this study was designed to evaluate the role of 5ALA in photodynamic therapy, fluorescence was reported from tumor areas. In this study, 5ALA was applied topically in a 20% solution to basal cell carcinoma (BCC) lesions. After waiting 3 h, the lesions underwent light exposure. Their initial results were promising. In the first 80 lesions that were treated using this technique, 90% of the lesions had a complete response rate and 7.5% had a partial response rate following a single treatment (40). Significant interest in the dermatologic utility of 5ALA has steadily grown since the work of Kennedy et al.

### Landmark 5ALA Studies in Dermatology

Szeimies et al. evaluated tissue localization of protoporphyrin (Pp IX) after topical application of ALA by measuring the fluorescence in different histological types of BCC lesions in 1994. These investigators used a 10% 5ALA and 10% CAB-OSIL M-5 (highly dispersed SiO2, CAB-OSIL Division, Cabot GmbH, Hanau, Germany) in propylene glycol ointment on fifteen patients with BCC lesions undergoing Mohs microsurgical resection (MMS). The area was bandaged after applying 5ALA ointment. Patients then underwent MMS 4–12 h after applying the 5ALA ointment. The control patients did not receive 5ALA prior to MMS. A microscope equipped with a 515–560 nm excitation filter was used during the MMS. The site of red fluorescence was compared to the histopathology. The results showed that tumors undergoing resection after waiting only 4 h had no significant fluorescence, whereas, tumors that underwent resection after 12 h showed a strong tumor-specific red fluorescence (10). These results differed from Kennedy et al. group who appreciated fluorescence of BCC tissue only after 3 h from the application of topical 5ALA. The optimal time required from the application of 5ALA to treatment was still unclear and further clinical studies were needed. The addition of solvents, such as Dimethyl sulfoxide (DMSO) and Ethylenediaminetetraacetic acid (EDTA) to the topical ointment was investigated by Peng et al. to determine if the use of these solvents can enhance the absorbability and specificity of 5ALA in tumor cells. They compared the topical application of 5ALA containing 20% 5ALA alone to 20% 5ALA plus 20% DMSO and 4% EDTA in patients with BCC lesions. They found that 5ALA alone was only located in the superficial layers in the lesion at 3 h post-application and both the penetration into the deeper parts of the lesion along with the degree of fluorescence was improved in the patients who received 20% 5ALA plus 20% DMSO and 4% EDTA after similarly evaluating lesions 3 h post-application. A 99% DMSO wash for 15 min prior to the application of 20% DMSO and 4% EDTA further enhanced the degree of penetration and fluorescence.

Despite apparent accumulation in tumors and improvement in surgical outcomes, 5ALA often showed poor delineation of tumors from normal tissue (41). Poor correlation often results from extension of the PpIX fluorescence beyond the true margins of the tumor and non-specific accumulation in benign lesions such as scars, sebaceous hyperplasia and others (42, 43). A few studies have shown that the accumulation of 5ALA in tumors with an intact epidermis which is usually seen in nodular BCC lesions is less compared to other types of BCC lesions that typical have epidermal ulcerations. This suggests that at the cellular level, the accumulation of 5ALA is not tumor specific but may be related to increased epidermal permeability and cellularity of tumors (44, 45). The use of esters of ALA, such as Methyl-ALA (MAL) improved delineation and specificity of the BCC lesions most likely secondary to the increased permeability of the molecule (43, 46, 47). Liposome encapsulation of 5ALA further enhances the lipophilicity of 5ALA and requires less time for maximal fluorescence (~2 h). Although better demarcation of non-melanotic skin lesions was found using liposome encapsulated 5ALA, the increased lipophilicity increases false positives due to the accumulation of Pp IX in sebaceous lesions, such as sebaceous hyperplasia along with viral warts, lichenoid inflammation and melanocytic nevi. Dense hairy areas also showed increased background fluorescence suggesting that this method is not suitable in regions with excess hair (42).

In addition to utility in BCC, topical 5ALA has also been found useful for the demarcation of squamous cell carcinomas (SCC) (48, 49). Delineation and complete excision of SCC lesions can be more challenging compared to BCC lesions due to its frequent irregular margins. Jeon et al. evaluated the use of 5ALA in delineating tumor margins in 64 patients undergoing MMS. Before the application of 5ALA, excessive crusts or scales were scraped off the lesion without causing bleeding and then the lesion cleaned with saline gauze. Nineteen patients received 20% 5ALA, 19 patients received 16% MAL and the remaining control patients did not receive a photosensitizer. The incubation period for the patients that received topical 5ALA was 6 h compared to 3 h for the patients received topical MAL. After the incubation period, a Wood lamp (ultraviolet examination light, model 31602, 356 nm; Burton Medical Products Corp., Chatsworth, CA) was used to determine the margins of the SCC lesion for MMS. The results showed that after MMS, the number of stages required for complete tumor removal was lower in the patients that received either 5ALA or MAL. There was no significant difference between 5ALA and MAL in terms of surgical benefits. A surgical benefit was not seen in all patients that had high-risk histologically SCC features, which may be due to irregular infiltrative patterns for these, PpIX may not penetrate the deeper layers and/or these cells do not produce as much PpIX compared to the cells with histologically low-risk SCC features (48).

### Appropriate 5ALA Dose and Route of Administration in Dermatology

As discussed above, topical use is the most common application of 5ALA in dermatology. Prior studies have used 5ALA or methyl ALA, mixed with DMSO/EDTA and or liposomal ALA. Further clinical studies, however, are warranted to determine the optimal clinical dermatology agent that should be used.

### Imaging 5ALA Fluorescence in Dermatology

Future development of imaging hardware and techniques to improve PpIX visualization and differentiation of abnormal tissue from normal tissue would be useful. Studies suggest auto-fluorescence is reduced within tumor cells with an excitation wavelength ~370 nm and an emission wavelength around 455 nm which is different compared to normal tissue (50–52). A few studies have shown improved 5ALA demarcation of BCC lesions when measured in concert with reduced background auto-fluorescence (50–52).

### Conclusions and Future Applications of 5ALA Fluorescence in Dermatology

**Figure 3:**
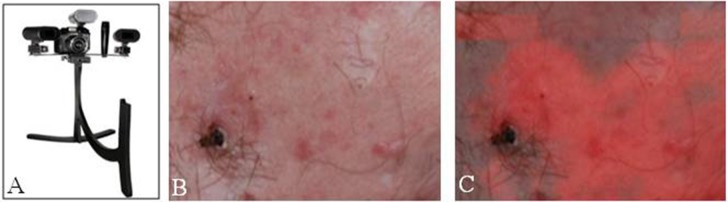
Fluorescence imaging in dermatology. **(A)** Clearstone UV-DA, DigiMed Systems-Medical digital imaging system with ability to take ultraviolet photos. Courtesy of DigiMed Systems. **(B)** Brightfield illumination vs. **(C)** fluorescence-overlay imaging of a facial basal cell carcinoma. [Intraoperative images courtesy of Wan et al. (53)].

Studies have shown improved surgical outcomes with the use of 5ALA in both photodynamic therapy and delineating tumor margins in MMS, especially in BCC lesions. Fewer studies to date have evaluated the use of 5ALA for SCC, though these studies have shown that 5ALA has potential for improve visualization of SCC. With a better understanding of the kinetics of 5ALA, along with advancements in imaging techniques, 5ALA-mediated visualization has potential for becoming standard practice in the treatment of BCC and SCC lesions, along with expanding use to other skin lesions such as melanoma (Figure 3).

## Obstetrics and Gynecology

### First Application of 5ALA Imaging in OB/GYN

Use of 5ALA in gynecologic cases now covers a broad spectrum of procedures (54–56) since PpIX was shown to accumulate in endometrial cancer cells by Wyss-Desserich and colleagues at non-toxic doses in 1996 (57). In this study, fluorescence was observed *in vitro* with best results found at 1 mg/ml incubated for 24 h and excited at 488 nm.

There is variation with the amount of 5ALA induced PpIX fluorescence in endometrium throughout the menstrual cycle. Highest fluorescence values are seen in secretory endometrium, followed by hyperplastic endometrium. In atrophic and proliferative phase endometrium fluorescence intensity and rate are the lowest (58).

### Landmark Studies and Appropriate 5ALA Dose/Route of Administration

Topical absorption of 5ALA is effective in patients with cervical neoplasia (CIN) II and III, as well as with lichen planus (55, 56). A cervical cap with 2–4 mL of 200 mg/ml 5ALA placed for 1.5 h demonstrates that dysplastic cervical tissue consistently has greater fluorescence than normal tissue. This persists at 1.5, 3, and 6 h after exposure (55). In Women with verified genital erosive lichen planus, 2 ml vaginal suppository of 6.25 mg/ml Hexyl 5-aminolevulinate applied for 3 h shows successful conversion and accumulation PpIX. Superficial fluorescence increases significantly in affected areas, and while this is not statistically significant at 30 min, it becomes so at 3 h. On microscopy, affected mucosa has strong fluorescence originating in submucosal inflammatory cells below the basal membrane (56).

Ovarian carcinoma is a good candidate for early detection with PpIX fluoroscopy as it commonly presents late in course and metastasizes. After initial tumor removal, second look operations can prevent recurrence. Intraperitoneal 5ALA solution given at a concentration of 30 mg/kg 5 h before laparoscopy has been evaluated as a route for administration and has shown systemic distribution comparable to oral/topical preparations. In a third of patients with metastases seen at second look operations, PpIX fluorescence was able to detect tumor in a majority of cases that would otherwise have been missed with brightfield illumination alone (59).

5ALA has also been used to delineate peritoneal endometriosis, occasionally as an incidental finding. At doses as low as 10 mg/kg administered orally 4–5 h before laparoscopy there is increased concentration of PpIX fluorescence in peritoneal lesions. These lesions are typically difficult to visualize. 5ALA utility in OB/GYN is limited by increased PpIX concentration in fimbriae and tubal tissue with unknown effects on fertility (54).

### Conclusion and Future Applications of 5ALA Fluorescence in OB/GYN

**Figure 4:**
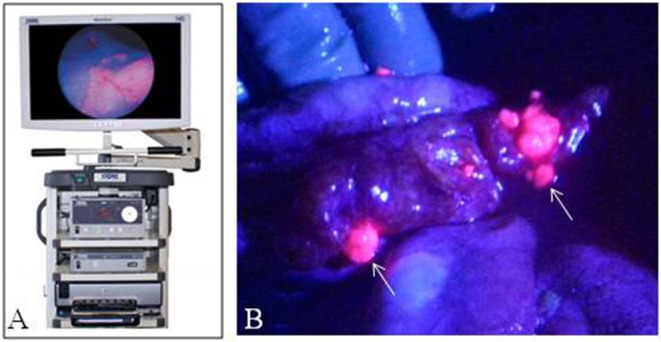
Fluorescence imaging in obstetrics and gynecology. **(A)** Karl Storz D-Light fluorescence endoscopy system. **(B)** Laparoscopic image of peritoneum with ovarian cancer metastases with fluorescence-overlay imaging, Sote small fluorescent metastases (arrows) were histologically consistent with tumor. [Intraoperative images courtesy of Yonemura et al. (60)].

Overall these early studies have shown 5ALA-mediated fluorescence in abnormal ovarian, endometrial, peritoneum and vulva tissue (Figure 4). There is potential for widespread and standardized use of 5ALA in OB/GYN especially as it pertains to second look operations in ovarian carcinoma. Further clinical studies are warranted to determine the safety and efficacy of 5ALA-mediated tumor visualization in OB/GYN.

## Head and Neck Surgery

### First Application of 5ALA in Head and Neck Surgery

Identifying innovative approaches to labeling and visualizing the borders of oropharynx and laryngeal neoplasms is a significant area of interest in head and neck surgery. The incidence of these neoplasms has increased during the last 2–3 decades secondary to alcohol and tobacco abuse (61). Studies show that survival rates increase with early stage carcinomas; however, the diagnosis of a tumor at the primary stage can be challenging. Earlier researchers used toluidine blue staining and auto-fluorescence imaging to visualize these lesions. However, Sabes et al. found a high false positive and false negative rate with the use of toluidine blue for detecting oral lesions which limited its widespread use. Similarly, Leunig et al. found that autofluorescence between normal and malignant tissue varied significantly between patients. Therefore, toluidine blue staining and autofluorescence never became popular in clinical use. Leunig et al. investigated the local use of 5ALA in patients with head and neck tumors. In this study, 16 patients with histologically confirmed oral SCC were given an oral rinse of 200 mg 5ALA dissolved in 50 ml of mineral oil. A zero- degree endoscope (Art, Nr. 7200 A; Storz, Tuttlingen, Germany) with ultraviolet light filtered xenon-arc lamp system along with an optimal multichannel analyzer (O-SMA 3; SI Instruments, Gilching, Germany) was used to measure the fluorescence contrast between tumor and normal tissue. In all patients, protoporphyin IX fluorescence was detected and significantly higher in the tumor compared to the surrounding healthy tissue. Therefore, the use of oral 5ALA potentially represented a new method for the early detection of oral dysplastic and malignant lesions (61). This study's promising results initiated further research in the use of 5ALA in head and neck surgery.

### Landmark 5ALA Studies in Head and Neck Surgery

Since the first paper evaluating the use of 5ALA in head and neck surgery in 1996, Leunig and other groups have continued to investigate the use of 5ALA in head and neck surgery. In 2000, Leunig et al. assessed the use of 5ALA for the detection of oral SCC in 58 patients. These patients rinsed with a 0.4% solution of 5ALA dissolved in mineral oil for 15 min while closely being supervised. After waiting for a 1 to 2.5 h period for incubation, biopsies were taken from red fluorescence areas presumed to represent tumor tissue along with tumor boundaries and normal tissue using a modified 0°degree endoscope equipped with a filter (OG515, Schott, Mainz, Germany). In this study, the topical use of 5 ALA had a sensitivity of 99% and a specificity of 60% in detecting oral squamous cell cancer and dysplasia with no direct side effects of 5 ALA. This study suggests 5ALA can be a useful tool in the early detection of oral malignancy, but further clinical studies were warranted (62). Zheng et al. later further evaluated the use of 5ALA in detecting oral cancer lesions. Their study included 49 patients with either clinically suspicious lesions or pathologically proven malignancies of the oral cavity. The patients rinsed with a 0.4% rinsing solution of 5 ALA dissolved in mineral oil for 15 min. After waiting for an incubation period of 1.5 to 2 h, all patients underwent fluorescence endoscopic-guided biopsies. The malignant and dysplastic histological findings closely correlated with PpIX fluorescence The sensitivity and specificity with the use of 5ALA for separating benign tissue from dysplasia was 92 and 96%, respectively. A sensitivity of 98% and a specificity of 96% was achieved in distinguishing carcinoma *in situ* from SCC with the use of 5ALA, and a sensitivity of 98% and specificity of 92% for distinguishing carcinoma *in situ* and squamous cell carcinoma from dysplasia was achieved with the use of 5ALA (63).

The use of 5ALA for distinguishing laryngeal CA from benign tissue has also been closely evaluated. Mehlmann et al. used a 5 ml 0.9% NaCl solution of 5ALA topically applied in 16 patients with suspected or histologically proven malignancies of the larynx via a nebulized inhaler 1–2 h prior to a microlarnyngoscopy. Microlaryngoscopy was performed through an optimized endoscope (Hopkins 0, Art. No. KSTEXB001-3 or 27005AI, Storz, Tuttlingen German) equipped with a special filter system (D-light, Art No. 20133201, Storz) that was attached to a footswitch that allowed switching between brightfield illumination and fluorescence imaging. Forty-five biopsies were taken. Areas of normal tissue appeared green in color whereas malignant sites showed a strong red fluorescence. The sensitivity and specificity of 5- ALA in separating normal tissue for malignant tissue was 95 and 80%, respectively (64). This was the first clinical study that showed 5ALA utility for the detection of laryngeal cancer. Csanady et al. later further investigated the use of 5ALA in detecting pharyngeal cancer. This study included 31 patients with either precancerous, malignant or benign lesions of the laryngeal or hypopharyngeal cavities. Patients received topical application of 1% 5ALA solution diluted with 0.9% saline solution via nebulizer. After a 1.5–2 h incubation period, fluorescence-guided endoscopic biopsies were performed. With the use of 5ALA, the sensitivity and specificity of detecting laryngeal and pharyngeal cancer from normal tissue was 96 and 76%, respectively. Hence, the laryngeal and hypopharyngeal tumors and their margins were found to be accurately outlined under fluorescence imaging showing the usefulness of 5ALA for intraoperative visualization of neoplastic tissue (65).

Visualizing the parathyroid gland while operating in a small space can be challenging, even for the most experienced surgeons (66). Methylene blue was initially used for identifying the parathyroid gland intraoperatively. However, its clinical use is limited as it is associated with serious side effects including: vascular pain, staining of the skin and urine, and neurological toxicity (66). This intrigued Prosst et al. to investigate the use of 5ALA in a 52 y.o female with refractory secondary hyperparathyroidism for identifying the parathyroid glands. The patient received 30 mg/kg of body weight of 5ALA dissolved in water given orally 4 h prior to surgery. After anterior cervical neck surgical exposure by an otolaryngologists, a 4 mm scope (30; Hopkins II; Karl Stortz CO) connected to D-light fluorescence system was brought into the operative field. Though the parathyroid glands were unable to be identified under white-light mode, they became clearly visible by their typical red fluorescence under 635 nm illumination. The patient did not experience any side effects from 5ALA (67). Suzuki et al. also investigated 5ALA's fluorescence in normal parathyroid glands in 13 patients with thyroid disease undergoing hemithyroidectomy. Patients were orally administered 20 mg/kg body weight of 5ALA dissolved in 10% glucose 5 h prior to surgery. After anterior neck exposure, the room was darkened and the incision was illuminated with a violet-blue light of 405 nm. In all 13 patients, parathyroid glands were easily identified by their red fluorescence, even when they could not be detected under normal light conditions (66). Takeuchi et al. further evaluated the use of 5ALA for identifying parathyroid gland tissue in 20 patients with primary hyperparathyroidism, 6 patients with secondary hyperparathyroidism, and 3 patients with thyroid tumors and normal parathyroid glands. All patients were administered oral 20 mg/kg 45 min to 5 h prior to surgery. In the majority of the cases, 5ALA accurately identified both normal and abnormal parathyroid glands (68).

### Appropriate 5ALA Dose and Route of Administration

The preferred application of 5ALA fluorescence during head and neck surgeries depends on the area of surgical interest. For oral lesions, a 0.4% oral solution of 5ALA dissolved in mineral oil rinsed for 15 min followed by 1 to 2.5 h incubation prior to illumination is the most common regime used (61–63). A 0.4–1.0% 5ALA solution diluted with 0.9% saline topically applied via a nebulized inhaler with an incubation period of 1–2 h prior to a microlarnyngoscopy has been reported for oral and pharyngeal lesions (64, 65). Whereas, for parathyroid and thyroid surgery, 20–30 mg/kg of 5ALA given orally with an incubation period between 45 min to 5 h has been reported (66–68). The safety of 5ALA, regardless of the administration route, has been questioned. Some studies suspected an increase in serum creatinine levels after 5ALA administration; however, this was refuted by Quon et al. when they concluded that the increased creatinine levels represented a false elevation due to substrate competition (69). Evidence in head and neck surgery shows that 5ALA is safe with little to no side effects, with the precaution to avoid sun exposure 24 to 48 h after ingesting 5ALA to avoid the risk of skin bleaching and other phototoxic effects on the skin and eyes (63, 70).

### Imaging 5ALA Fluorescence in Head and Neck Surgery

At the molecular level, 5ALA is metabolized by neoplastic or highly metabolic tissues into protoporphyrin IX, a photosensitive metabolite that is excited between wavelengths 375–440 nm and subsequently emits fluorescence between 635 and 700n m (71). The use of an endoscope equipped with a special filter system attached to a footswitch that allows changing between brightfield illumination and fluorescence excitation is the preferred method for detecting the red fluorescence when 5ALA is applied in head and neck surgery.

### Conclusions and Future Applications of 5ALA in Head and Neck Surgery

The above studies suggest that 5ALA has potential for improving the early detection of suspected oral and pharyngeal cancerous lesions and may reduce operative time and rate of reoperation in patients with parathyroid and thyroid disease. The disadvantage of 5ALA is the need for patients to avoid sun exposure 24 to 48 h after exposure to 5ALA. More clinical studies are needed to further validate the surgical benefits of 5ALA compared to routine surgery before the use of 5ALA can become FDA-approved for clinical practice in head and neck surgery.

## Gastrointestinal Surgery

### First Application of 5ALA in Gastrointestinal (GI) Surgery

The use of 5ALA for photodiagnosis in gastroenterology and gastrointestinal surgery, while broad and encompassing esophageal, gastric, hepatic, and colorectal pathologies, has not yet replaced established standard practice in this field. Interestingly, its first use was aimed at a predominantly preventive strategy during screening colonoscopies to identify mucosal adenomas with malignant potential. Although adenomas are benign, there is no method to distinguish between benign and malignant lesions by conventional colonoscopy. Therefore, Eker et al. (72) described the use of 5ALA to aid in the differentiation of mucosal tissue, potentially as a means to decrease cost and labor involved in removing benign lesions. Of 57 selected patients undergoing laser-induced fluorescence colonoscopy, 41 were ultimately included. Patients were given oral ALA at 5 mg/kg and underwent the study after 2–3 h. A nitrogen laser was tested at excitation wavelengths of 337, 504, and 436 nm. The excitation of tissues at 337 nm was used, as that wavelength yielded the maximum difference in emission between normal and adenomatous tissue. All patient groups were combined at the 337 nm wavelength, including patients who were treated with 5ALA and those who were not. When excited at 337 nm, the sensitivity for adenoma detection was 100% and the specificity was 96%. At the other wavelengths of 405 and 463 nm, the sensitivity and specificity were also higher for 5ALA-treated patients than those without.

The application of 5ALA in predicting the malignant potential of dysplastic epithelial cells associated with ulcerative colitis also demonstrated an efficacious use of the fluorescent molecule. One group evaluated the use of 5ALA to enhance the detection of dysplastic tissue that may otherwise be missed from the conventional standard of care, which is four-quadrant random colon biopsies. In this study, 37 patients underwent 54 colonoscopies and received systemic oral ingestion, a local enema, or a local catheter-directed spray form of 5ALA. The local spray had the highest sensitivity of 100% for detecting malignant tissue. Overall, this serves as a promising modality to increase detection of dysplasia with a reduction of sampling error and unnecessary random biopsies (73).

### Landmark 5ALA Studies in GI Surgery

**Figure 5:**
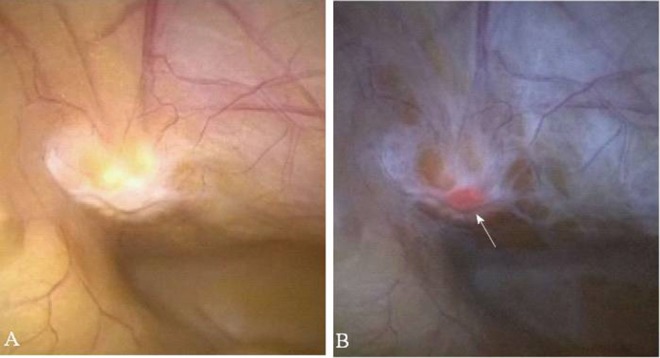
Fluorescence imaging in Gastrointestinal Surgery. **(A)** Non-specific peritoneal area under white light illumination. **(B)** Corresponding fluorescence overlay image shows fluorescence foci histologically consistent with metastatic gastric cancer (arrow). Images obtained with Karl Storz D-light endoscope system. [Intraoperative images courtesy of Namikawa et al. (74)].

Similar to its use for colorectal cancer, endoscopic 5ALA fluorescence has been explored as a surveillance method for detecting dysplastic, pre-malignant tissue of the esophagus (Barret's), and more recently, assisting in laparoscopy-guided resection of gastric and hepatic malignancies (Figure 5). These studies have highlighted the growing number of 5ALA applications in GI surgery while also demonstrating the mild, transient adverse reaction profile with oral administration.

Much of the data pertaining to the use of 5ALA is for the diagnosis and detection of dysplastic tissue in Barrett's esophagus during upper endoscopy. Barrett's esophagus is the replacement of normal esophageal epithelium (stratified squamous) to colonic epithelium (simple columnar), typically due to chronic exposure to acid reflux from the stomach. It is considered a premalignant lesion and requires endoscopic surveillance with biopsy. The biopsy for Barrett's esophagus is extensive: it involves obtaining 4 random quadrant biopsies for every 2 cm length of the Barrett's esophagus. The tissue within Barrett's esophagus can be classified as a range: nondysplastic, low grade dysplasia, high grade dysplasia, to adenocarcinoma, but these cannot be distinguished by gross examination with standard endoscopy. Given the time and cost required to biopsy Barrett's esophagus via the conventional method, alternative methods are investigated to better pinpoint dysplastic lesions, such as the use of ALA.

Brand et al. (75) evaluated the use of 5ALA to identify dysplasia within Barrett's esophagus, high grade dysplasia in particular. Twenty patients with known Barrett's esophagus were given 5ALA at 10 mg/kg dissolved in orange juice 3 h prior to endoscopy. A nitrogen laser at excitation of 400 nm was used at a total of 97 mucosal sites. The use of 5ALA provided detection of high grade dysplasia from normal tissue at 77% sensitivity and 71% specificity. In nodular high grade dysplasia the sensitivity and specificity of 5ALA-mediated photodiagnosis approaches 100%. Although the use of fluorescence endoscopy may assist in identification of these high grade lesions, there is no definitive data that suggest its use is superior to random biopsies.

Stepinac et al. (76) performed a similar study investigating the fluorescence detection of dysplasia and early cancer in Barrett's esophagus using 20 mg/kg of 5ALA vs. conventional random four-quadrant biopsy. Twenty-eight patients with known Barrett's esophagus were selected for the study. All patients first underwent conventional endoscopy with random four quadrant biopsies at 1 cm intervals approximately 4–6 weeks prior to fluorescence endoscopy. A total of 532 biopsies were taken. During fluorescence endoscopy, a total of 178 biopsies were taken, 81 in fluorescent regions and 97 in non-fluorescent regions. The sensitivity and specificity of fluorescence-guided biopsies were 100 and 63%, respectively. Fluorescence endoscopy identified 5 low grade dysplasia lesions that were not identified by conventional biopsy. Conventional biopsy identified 3 low grade dysplasia lesions that were not detected during fluorescence endoscopy. Both the conventional and 5ALA methods were able to identify high grade dysplasia and adenocarcinoma, but neither is ideal for identification of low grade dysplasia. However, the use of ALA and fluorescence endoscopy may result in fewer biopsies needed to achieve the same diagnosis (81 biopsies vs. 531).

Although the incidence of gastric cancer has decreased overall, it remains one of the most common cancers worldwide. It commonly spreads by peritoneal seeding. The presence of peritoneal dissemination can alter the treatment decision-making between surgery and chemotherapy. In patients with locally advanced disease, performing a staging laparoscopy can help guide treatment. Staging laparoscopy has a sensitivity of ~30–40% for peritoneal seeding. Kishi et al. (77) has evaluated the use of 5ALA to enhance the tumor detection rate in advanced gastric cancer. Thirteen patients with known gastric cancer were selected to undergo staging laparoscopy. They are given 1 g 5ALA 4 h prior to laparoscopy. The detection rate for metastatic lesions using 5ALA had a sensitivity of 93% and a specificity of 100%. However, there were inconsistencies in the detection of the primary tumors. Although all primary tumors with serosal invasion were detected, 5ALA failed to help identify two primary tumors without serosal invasion and two primary tumors with adjacent organ invasion. This emphasizes the limitation of the use of visible wavelength fluorescence imaging, for while it is useful in detecting surface tumors, it may not visualize tumors that are deeply embedded in tissue.

In 2017, Ushimaru et al. (78) performed 5ALA-assisted staging laparoscopy on 113 patients with advanced gastric cancer and based their treatment strategies on the results of the laparoscopy. They retrospectively evaluated the outcomes, and identified predictive factors for peritoneal metastasis. All patients were given 1 g 5ALA 3–5 h prior to laparoscopy. Observation of abdominal cavity using white light and blue light was done using the Storz D-light System laparoscope. Patients noted to have peritoneal metastasis and/or positive cytology, as diagnosed by 5ALA staging laparoscopy, underwent chemotherapy and an interval gastrectomy if a later laparoscopy revealed negative peritoneal disease. Those without metastasis or cytology underwent definitive gastrectomy during the same procedure. Patients with no peritoneal metastasis had a similar estimated survival than patients with peritoneal metastasis only seen with 5ALA. Factors that were predictive for metastasis were advanced T stage, diffuse-type histology, ascites, and female sex. This study suggests that the use of staging laparoscopy with 5ALA may enhance the diagnostic accuracy for advanced gastric cancer patients and thereby improve their overall treatment outcome.

5ALA-mediated photodiagnosis of hepatic tumors is currently being explored. Kaibori et al. (79) evaluated a total of 134 patients undergoing hepatic resections. They compared the utility of both 5ALA and Indocyanine Green for the intraoperative detection of tumors. Although Indocyanine Green had higher sensitivity of tumor detection (96% vs. 57%), the specificity of detection by 5ALA was 100%. One limitation of photodiagnosis for solid organ tumors is short distance of penetration. 5ALA mediated fluorescence only penetrates 2–5 mm of tissue, limiting its use to superficial tumors. The adverse effects, while transient and self-limited, may also deter patient participation.

A serious complication of hepatic resection is postoperative bile leak. Inoue et al. (80) evaluated 737 patients who underwent hepatic resections. Of these, 109 patients were given oral 1 g 5ALA dissolved in 20 ml of 50% glucose approximately 3 h prior to hepatic resection, which is excreted in the bile. Intraoperatively, patients were evaluated for leaks after resection, first by gross evaluation then by blue light (405 nm) illumination. Fluorescence imaging using 5ALA increased the intraoperative detection rate of bile leaks from 8.3 to 13.7%. Given the mildly increased detection rate, more studies will be needed to justify routine use of 5ALA.

### Appropriate Dose of 5ALA and Route of Administration

The optimal dosing of 5ALA in the upper and lower GI tract did not vary significantly from that which was historically used in non-GI related malignancies. Since its earlier application for the photodiagnosis of colorectal (72, 73) and esophageal carcinoma (75, 76), oral administration of 5 and 10 mg/kg, respectively, demonstrated superior sensitivity and specificity in identifying neoplastic tissue on par with that of tradition biopsy-based surveillance methods. Oral administration of even greater amounts of 5ALA up to 1 g for its more recent gastric and hepatic applications (77–80), including the fluorescence of post-resection bile leaks, has shown equal efficacy.

### Conclusions and Future Applications of 5ALA in GI Surgery

Although there is promising data regarding the use of photodiagnosis in gastroenterology and general surgery, there lacks conclusive data to push its use into standard practice. More randomized control trials with larger patient samples are still needed at this time.

## Cardiothoracic Surgery Section

### First Application of 5ALA in Cardiothoracic Surgery

The first study examining 5ALA's ability to detect early stage lung cancer was published in 1996 by Baumgartner et al. (81). In this study, Baumgartner administered 5–10 wt% (250–500 mg 5ALA in 0.5 ml isotonic saline) through a medical nebulizer to 7 patients with positive or suspicious sputum cytology, but negative white light bronchoscopy, for inhalation for 30–40 min followed by fluorescence bronchoscopy after 3 h. An ultraviolet light with 406.7–413.5 nm was used for excitation. A target integrating color CCD video camera was adapted to the bronchoscope and captured emission wavelength of 635 nm and 705 nm. Baumgartner calculated that the amount of 5ALA inhaled within lung tissue was independent of patients' breathing patterns. Thus, patients can have a wide range of pulmonary function without concern for variability in 5ALA concentration. A peak plasma blood PpIX concentration was found at 3.5 h after inhalation. No side-effects were observed in the 7 patients. A total of 30 tissue samples were taken which showed nine tumors, including seven carcinoma *in situ*, by PpIX fluorescence. Sensitivity was 100% and specificity ranged from 30 to 50% due to five tissue samples exhibiting weak fluorescence with indeterminate diagnoses. This study showed that 5ALA inhalation was safe, could mediate detection of carcinoma *in situ* within lung tissue, and may be used to assist diagnosing early bronchial malignancies.

### Follow-Up Study Using Inhaled 5ALA for Bronchial Tumors

Following Baumgartner's study of 7 patients, Piotrowski et al. performed a perspective feasibility study on safety and efficacy of 5ALA for diagnosing bronchial neoplasms (82). Forty-nine patients, divided into four groups, inhaled either 5 or 10 ml of 5ALA solution 3 h before bronchoscopy examination. Xenon short arc lamp with a special filter system was used for fluorescence excitation (λex = 375–440 nm) along with an integrated filter that blocked remitted excitation (long pass = 440 nm). Groups consisted of laryngo-tracheo-bronchial tumors that were previously diagnosed by conventional diagnostic modes (*n* = 17); patients with prior surgery due to bronchial tumors (*n* = 6), patients with prior surgery for laryngeal cancer (*n* = 4); and present or ex-heavy smokers without signs of tumor in conventional examinations (*n* = 22). There was no significant difference between pre and post FEV1 values. The overall sensitivity of 5ALA was 82% and specificity was 62% for all groups, PPV of 45% and NPV of 90%. When brightfield illumination (WLB) plus 5ALA was compared with brightfield alone, there was an increase in sensitivity by 2.1% and NPV by 6%, but decreased specificity by 35.4% and PPV by 53.1%. When the heavy smoker group was excluded (due to increased number of false positives), the WLB+5ALA sensitivity increased by 22% and NPV by 34%, whereas specificity decreased by 26% and PPV by 35%. Due to its higher sensitivity, 5ALA was able to identify recurrent SCC in one patient and a synchronous lesion in another patient that were WLB-negative. Because of the high number of false positive samples, authors concluded that 5ALA should not be used for screening. Rather in combination with WLB, it could be used to guide the physician in choosing proper sites for biopsy due to its higher sensitivity and NPV. Additionally, in this study 5ALA induced transient bronchial obstruction in 2 patients from the heavy smokers group that was reversed with short acting beta agonists.

### 5ALA Fluorescence for Staging Pleural Malignancies in Patients With Inconclusive Pleural Effusions

Thoracoscopy is often used in patients with negative pleural fluid examinations for staging of malignancies. However, some cases still remain undiagnosed or understaged (83). In 2006, Baas et al. in a feasibility study, incorporated fluorescence and white light inspection on 26 patients with non-diagnostic pleural effusions to test 5ALA's efficacy in diagnosing and staging of pleural malignancies. Three patients were excluded either due to multiple adhesions within thoracic cavity or inability to perform endoscopic inspection. Patients ingested a 2,000 to 2,500 mg capsule of 5ALA depending on body weight followed by thoracoscopy 3–4 h later. Fluorescence images were recorded using the D-LIGHT System with <500 nm for excitation and long-pass filter (470 nm long-pass). In 23 patients, a total of 111 biopsies were taken. Fifteen patients were diagnosed with malignant mesothelioma, 5 with metastases. Three patients had benign plaques and one with empyema. One patient who did not receive initial diagnosis developed mesothelioma 6 months later. A discrepancy between white light and 5ALA occurred in 37 biopsies and further analysis showed there was no improvement using 5ALA to obtain diagnosis. However, with 5ALA, 4 patients upstaged their diagnosis through detection of small lesions (<3 mm) throughout the parietal and visceral pleura as well as the collapsed lung. Complications occurred in 3 patients from thoracoscopy. No side-effects were reported using 5ALA besides grade 1 skin burn in 3 patients 28 to 36 h after 5ALA intake. Authors showed that 5ALA can help improve staging in patients with pleural malignancy and could help guide in choosing proper biopsy sites during thoracoscopy.

In another study of undiagnosed pleural effusions by Pikin et al. 23 patients with non-conclusive pleural effusions received 25 mg/kg of 5ALA 3 h before video-assisted bronchoscopy (84). A total of 118 biopsies were taken. Both white light and fluorescence thoracoscopy detected pleural deposits in 20 patients but fluorescence was able to detect additional lesions in 12 of the 20 patients. In three other patients with macroscopically normal pleura by white-light mode, 5ALA detected micrometastases in one patient that was metastatic lung adenocarcinoma by histological examination. Results from combined conventional and florescence thoracoscopy showed specificity of 88.4%, sensitivity of 89.1% and diagnostic accuracy of 88.9%—results much higher than other studies. Authors pointed out that detecting additional lesions in patients with macroscopic pleural spread does not influence outcome in the majority of cases. However, detection of pleural micometastases in patients with peripheral lung cancer and visceral pleural invasion could improve pre-operative approaches and possible outcomes.

### Pharmacokinetics of Inhaled 5ALA for Optimum 5ALA-Induced Protoporphyrin IX Fluorescence in Bronchial Tissue

In Baumgartner et al.'s 1996 study, he first showed that 5ALA was safe for inhalation. In a follow-up study, Hautmann et al. examined *in vivo* kinetics of inhaled 5ALA that generate the greatest difference in fluorescence between tumor and adjacent bronchial tissue. Nineteen patients with known or suspected bronchial carcinoma are given 200 mg of 5ALA dissolved in 5 ml of isotonic NaCl via inhalation. Patients are then randomized to 1,2,3,4, or 6 h before bronchoscopy under local anesthesia.

Excitation wavelength of 380–440 nm was used and emission greater 630 nm, with a peak emission at 635, were measured using Optical Multichannel Analyzer (OMA). Spectroscopy was then analyzed on all macroscopically suspicious areas and areas showing porphyrin fluorescence. A total of 38 biopsies were taken that showed sensitivity that is almost twice that of white light, but with a significant decrease in specificity. This decrease in specificity was explained by the uptake of 5ALA by inflammatory lesions. Spectroscopy showed that normal tissue showed a maximum fluorescence 200 min after 5ALA application and lesions with moderate dysplasia at 160 min after 5ALA application. The spectral data showed significant difference between lesions with moderate dysplasia and normal, as well as lesions with moderate dysplasia and lesions with mild dysplasia 80 to 270 min after 5ALA inhalation. During this time interval, 5ALA fluorescence in lesions with moderate dysplasia can exhibit fluorescence values 5 times higher compared to the normal tissue. No difference was seen in lesions with mild dysplasia and normal tissue (85).

### 5ALA Facilitates Differentiation of Primary Lung Cancer With Pleural Invasion

Kitada et al. recruited a total of 40 patients diagnosed pre-operatively with lung cancer to undergo white light and 5ALA-mediated photodiagnosis. Patients consisted of 28 cases with primary lung cancer, 8 with metastatic lung tumors, 2 with malignant pleural tumors, and 2 with benign tumors. All lung metastases on the pleural surface, pleural malignant mesotheliomas and benign tumors were visualized under red fluorescence. For primary lung tumors, red fluorescence was confirmed in 15 of 28 patients (53.5%). All P11–P13 (ranging from tumor invading beyond elastic layer to tumors invading parietal pleura) tumors were visualized (10/10). However, visualization decreased to 5/18 (27.7%) for p10 cases (tumor within subpleural parenchyma or superficial invasion of pleural connective tissue). These 5 cases had been previously diagnosed as p11. Authors showed that 5ALA enhances accurate diagnosis of malignant lesions on the pleural surface as well as detection and localization of small disseminated lesions and small metastatic tumors to the lung (86).

### Current Status and Future Directions of 5ALA in Cardiothoracic Surgery

Currently, 5ALA is still used as a research tool for photodynamic diagnosis in cardiothoracic surgery. Future direction of 5ALA includes a possible of direct comparison of brightfield vs. 5ALA-mediated fluorescence bronchoscopy in a randomized control trial to determine which yields higher sensitivity and/or specificity.

### Conclusion

Clinical studies show 5ALA photodynamic diagnosis yields higher sensitivity but lower specificity in identifying lung and pleural malignancies. When added with conventional brightfield illumination, 5ALA can help visualize small primary tumors (<3 mm), small lung metastases and primary lung cancer with pleural invasions. 5ALA may prove useful for guiding surgeons to specific biopsy sites and in the upstaging of tumors.

## Discussion

The clinical use of fluorescent molecules dates back to the start of the twentieth century. Coined “Photodynamic Wirkung,” or photodynamic phenomenon, European scientists first described how to utilize these molecules to macroscopically label abnormal tissue (87). While initially a conceptual application, this methodology has driven numerous oncologic investigations of naturally derived fluorescent molecules. Hematoporphyrin derivatives were introduced in the mid-twentieth century as potentially valuable diagnostic tools and treatment modalities. Early studies conducted by Lipson and colleagues (88) using rudimentary, yet novel, endoscopic devices to differentiate neoplastic cells from normal tissue further developed our understanding of hematoporphyrin derivatives in the surgical setting. Currently FDA-approved for its use is glioma surgery, 5ALA has undergone significant advances in its applications to include neurosurgical, head and neck, urological, cardiothoracic, gastrointestinal, and OB/GYN surgery. This historical review of clinical studies highlights the rapidly expanding role of 5ALA in the diagnosis and treatment of neoplastic disease.

Within each surgical field, studies have outlined the advantages and disadvantages 5ALA-mediated photodiagnosis. Due to its ubiquity in the heme synthesis pathway of all cells, and preferential accumulation of PpIX within neoplastic cells, the route and dosage of 5ALA has minimally varied. Intra-venous, oral, intra-peritoneal, intra-vaginal, inhaled, and topically administered 5ALA all demonstrate a similar optimal dosing (5–30 mg/kg), and often, dose-dependent responses. For many 5ALA applications, maximum dose are determined not by increased adverse reactions, but rather plateauing of sensitivity and specificity in neoplastic cell labeling. This phenomenon seems intuitive given 5ALA's role as a naturally occurring PpIX precursor. Studies have noted relatively benign and avoidable adverse reactions including bronchospasm with inhaled variants, and photosensitivity most prevalent with topical and oral administration of 5ALA. Regardless of route of administration, the sensitivity of 5ALA photodiagnosis has varied from 83% with low doses in urologic dysplasia to 100% for most other applications. Albeit rare, a notable disadvantage of using 5ALA is represented in its poor specificity in differentiating moderate dysplasia or cells exhibiting inflammatory changes from normal tissue or higher-grade dysplastic lesions. This limitation is most apparent in labeling urothelial carcinoma and BCC/SCC, resulting in high false positive rates due to epithelial hypercellularity or inadequate depth of topical penetration, respectively. In order to ameliorate non-specific labeling observed with these applications, the composition of 5ALA delivery systems (liposomal) and solvents (EDTA or DMSO) are continuing to be evaluated.

Imaging devices used to visualize 5ALA uptake and PpIX fluorescence are also advancing in their design and implementation. In early clinical applications of hematoporphyrin derivatives, such as PpIX, for malignancies of the upper GI and cardiopulmonary systems, a 400-watt mercury lamp transmitted white light via glass fiber cables through which excitatory (~400 nm) wavelengths were separated by a quartz rod placed in a rigid bronchoscope (88). Using optical filters in the form of glasses or eye-shields, the surgeon would then visualize only red-fluorescence wavelengths corresponding to PpIX. Current-day fluorescence-guided surgery using commercially available wide-field microscopes is significantly less cumbersome and utilizes more sensitive short pass and long pass filters integrated within the system. Additional imaging hardware designs have aimed to improve on the historical limitations of using 5ALA in its various surgical contexts. While still investigational, newer imaging hardware optimizes the resolution of PpIX in either islands of neoplastic cells or areas of lower-grade lesions where PpIX fluorescence may not be as robust as that of higher-grade lesions. The largest obstacles toward this goal have been to mitigate signal-to-noise ratio of autofluorescent tissue and to achieve a greater sensitivity in localizing deep, labeled tissue (89).

Administration of 5ALA has granted surgeons within multiple subspecialties the ability to more accurately visualize malignant tissue during surgery. The vast data from studies collected during the past 6–7 decades is representative of 5ALA's small side effect profile and reliable efficacy. The growth of 5ALA's intraoperative applications during this timeframe has been paralleled by advancements in imaging technology focused on improving PpIX visualization. These clinical trials suggest 5ALA is a relatively safe molecule for generating intraoperative photodiagnosis of malignant tissues across multiple surgical-oncology subspecialties.

## Author Contributions

JG, AV, HW, AB, MK, SC, ZA-A, MB, HO, JI, SYu, and CL wrote key portions of the manuscript and created the figures. DA, JL, PN, KB, and SYo oversaw the writing process, provided mentorship, edited, and contributed to manuscript.

### Conflict of Interest Statement

The authors declare that the research was conducted in the absence of any commercial or financial relationships that could be construed as a potential conflict of interest.
